# An acoustic signature of extreme failure on model granular materials

**DOI:** 10.1038/s41598-022-20231-6

**Published:** 2022-10-31

**Authors:** T. T. T. Nguyên, T. Doanh, A. Le Bot, D. Dalmas

**Affiliations:** 1 Ecole Nationale des Travaux Publics de l’Etat, LTDS (UMR 5513), Vaulx en Velin, France; 2 Ecole Centrale de Lyon, LTDS (UMR 5513), Ecully, France

**Keywords:** Applied physics, Experimental particle physics

## Abstract

Unexpectedly, granular materials can fail, the structure even destroyed, spontaneously in simple isotropic compression with stick-slip-like frictional behaviour. This extreme behaviour is conceptually impossible for saturated two-phase assembly in classical granular physics. Furthermore, the triggering mechanisms of these laboratory events remain mysterious, as in natural earthquakes. Here, we report a new interpretation of these failures in under-explored isotropic compression using the time-frequency analysis of Cauchy continuous wavelet transform of acoustic emissions and multiphysics numerical simulations. Wavelet transformation techniques can give insights into the temporal evolution of the state of granular materials en route to failure and offer a plausible explanation of the distinctive hearing sound of the stick-slip phenomenon. We also extend the traditional statistical seismic Gutenberg–Richter power-law behaviour for hypothetical biggest earthquakes based on the mechanisms of stick-slip frictional instability, using very large artificial isotropic labquakes and the ultimate unpredictable liquefaction failure.

## Introduction

How to understand the natural earthquakes and even predict theirs occurrences, beyond the common initial proposal of abrupt release of slowly accumulated energy in the early days of geophysics^1,2^? These questions are notoriously difficult with no clear answers for scientists to date, despite an ever growing amount of theoretical and experimental studies^3,4^. Laboratory experiments have logged years of experience searching for the inner workings of artificial earthquakes on small scale using model granular materials in thin layers as proxies for fault zones in real earthquakes^5–7^. Can we extend the traditional empirical power-law of energy-frequency distribution in seismicity using these extreme laboratory quakes? Passive recordings of acoustic emissions offer an attractive non-invasive method to access the changing state of the testing system trying to unlock the still mysterious triggering mechanisms of labquakes, and natural ones^8,9^. The density of vibrational modes *D*(*f*, *t*) of frequency *f* and time *t* of the velocity auto-correlation function is a first practical proposal^9,10^.

The continuous wavelet transform $T_{\psi }(f,t)$ provides a simpler alternative to *D*(*f*, *t*) without the arbitrary fixed temporal window for the time-frequency analysis of *D*(*f*, *t*), and offers highly more resolved details on the characteristics of the signal. The $T_{\psi }(f,t)$ of a continuous temporal signal $u(\tau )$ by a mother wavelet $\psi$ at frequency *f* (analogous to wavelet scale parameter *s*) and time *t* is defined as the inner product of the signal *u* with wavelet $\psi$^11^:

1 $$\begin{aligned} T_{\psi }(f,t) = T_{\psi }[u](f,t) = \frac{1}{\sqrt{f}} \int \limits _{-\infty }^{\infty } u(\tau ) {\overline{\psi }} \Big (\frac{\tau -t}{f}\Big ) d\tau \end{aligned}$$

${\overline{\psi }}$ is the complex conjugate of $\psi$ shifted in time by *t* and scaled by *f* to match the time-continuous signal $u(\tau )$. We use the Cauchy complex fractional function as progressive mother wavelet $\psi _{n}$ due to its excellent time-frequency localization and its fast computation time^12,13^.

2 $$\begin{aligned} \psi _{n} (x) = \Big (\frac{i}{x+i}\Big )^{n+1} \end{aligned}$$

where $i^2 = -1$ and *n* a dimensionless order parameter. This accurate time localization of the instantaneous dominant frequency is better than that of Short-Time Fast Fourier Transform.

In this paper, we report the unusual stick-slip-like failure behaviour of saturated and loose granular materials in very simple isotropic compression, without shear stress, as simplest laboratory tests representative of catastrophic natural earthquakes^14^. We consistently use $T_{\psi }(f,t)$ of acoustic emissions and multiphysics numerical simulations to offer a new plausible interpretation of the extreme frictional failure of a granular system, especially the surprising audible sound accompanying every stick-slip event. We also extend the traditional statistical Gutenberg–Richter power-law in seismicity for potentially largest earthquakes, based on the energy-frequency distribution of very large isotropic instability events, including the ultimate isotropic liquefaction failure for the first time.

**Figure 1:**
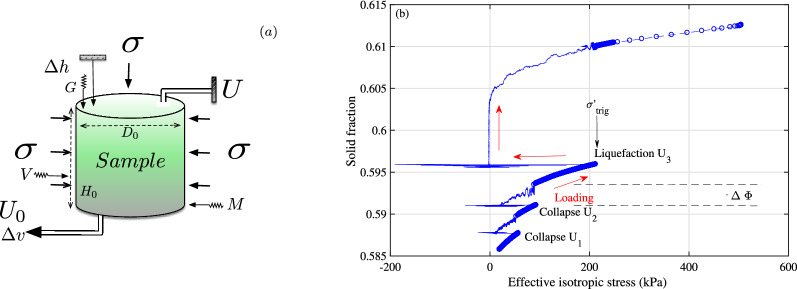
Dynamic instabilities of model granular materials in drained isotropic compression. (**a**) Sketch of the experimental setup of the drained isotropic compression on cylindrical sample inside a triaxial cell. The axial displacement $\Delta h$ and the water volume $\Delta v$ were measured to estimate the global axial strain $\varepsilon _{a}$ and solid fraction $\Phi$. The back pressure $U_0$ was applied at the sample bottom, while the measurements of static pore-water pressure *U* at the sample top permit to assess the homogeneity of the effective stress state. One piezoelectric accelerometer measures the vertical acceleration *G* of the sample top cap. One complementary non-contact portable laser vibrometer *V* and one microphone *M* record the lateral surface vibration and the radial sound pressure outside the triaxial chamber. (**b**) Collapses $\hbox {U}_1$, $\hbox {U}_2$ and liquefaction $\hbox {U}_3$ in isotropic drained compression from 20 up to 212 kPa of confining pressure. Red arrows denote the direction of loading.

## Experimental setup

We study the isotropic compression on a short 3D cylindrical sample, $H_{0}$ = 70 mm in height and $D_{0}$ = 70 mm in diameter, of small soda-lime glass beads, with mean grain diameter, $D_{50}$ = 0.723 mm, and mean surface roughness *Sa*, the arithmetic mean height, of only 36 nm^15^, inside a triaxial cell (Fig. 1a). The granular materials, of about 1.23$\times 10^{6 }$ quasi-perfect spherical grains, are placed inside an open ended cylindrical latex membrane of 0.3 mm of thickness. The fully water-saturated (no air pores) and very loose sample is slowly compressed under stress-controlled mode with constant stress rate around 1 kPa/s to have a quasi-static regime under low dimensionless inertial number $I \approx$ 10$^{-6}$^14^ in drained loading condition (constant pore fluid pressure). One tiny piezoelectric accelerometer measures the instantaneous vertical acceleration of the sample top cap. One complementary non-contact portable laser vibrometer and one free-field prepolarized microphone measure the local lateral surface vibration and the radial sound pressure outside the triaxial chamber. Two LVDT sensors record the sample axial and volumetric changes, $\Delta h$ and $\Delta v$, to estimate the global axial strain $\varepsilon _{a} = \Delta h / H_{0}$, the global volumetric strain $\varepsilon _{v} = \Delta v / V_{0}$ and the global solid fraction $\Phi$ (the volume of solids to the total volume) during the isotropic loading (details in^16,17^).

**Figure 2:**
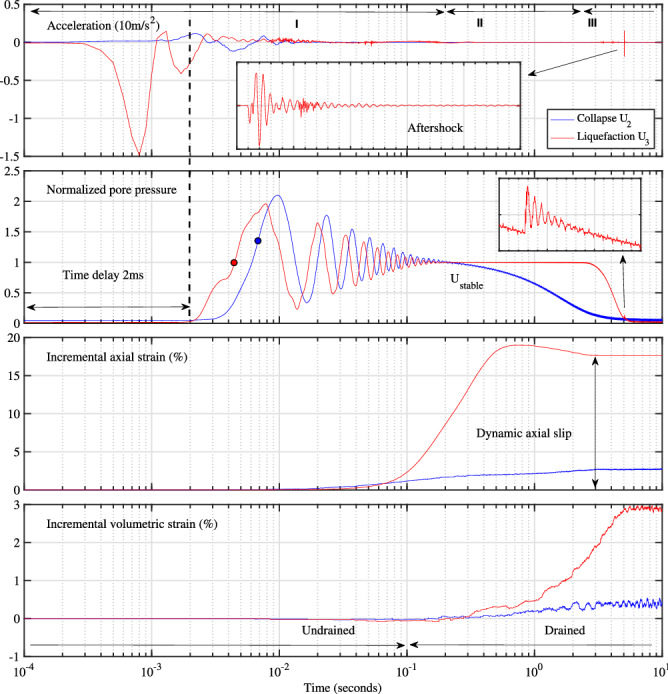
Typical temporal evolution of isotropic collapse $\hbox {U}_2$ (blue) and liquefaction $\hbox {U}_3$ (red) on 0.7 mm CVP beads in isotropic drained compression: successively vertical top cap acceleration, normalised excess pore pressure, permanent incremental axial and volumetric strain. The superimposed liquefaction points (solid circles) on normalised excess pore pressure, with systematic time delay (vertical black dashed line), are above the unity level, indicating non-liquefaction event for collapse $\hbox {U}_2$. The axial strain of collapse event is magnified by a factor of 30. A small aftershock (inset figures), without affecting the liquefaction results, occurs after 5 s.

## Results

Instead of having a usual continuous increase of solid fraction $\Phi$ with increasing internal effective stress $\sigma '$^18^ of real granular media such as natural sands^14^, the isotropic drained compression creates irregular stick-slip-like events termed as isotropic local collapses $\hbox {U}_1$, $\hbox {U}_2$^16,19^ in a sawtooth behaviour in Fig. 1, beginning from 20 kPa up to an unexpected full destruction $\hbox {U}_3$ of the granular structure at 212 kPa. The compressibility behaviour presents three spontaneous events occurring successively at uncontrollable triggering stress ${\sigma '}_{trig}$ of 57, 93 and 212 kPa. Each event begins systematically by a very fast change of the vertical top cap acceleration and of the internal stress, Fig. 2, in logarithmic scale to emphasize the suddenness in behavioral changes in the millisecond scale (details in Supplementary Materials S1). Remind that the cell pressure remains constant within 0.1 kPa during the whole instability motion without any visible macroscopic shear stress^17^. The final $\Phi$ approaches an asymptotic value of 0.615, which is still far below the maximum packing fraction (0.639) for an assembly of spherical particles^20^. Figure S1 gives the complementary temporal evolution of the acceleration of the second event $\hbox {U}_{2}$ in the normal linear scale of 200 s, showing clearly the quasi-static regime under all-round compression below 0.002 *g*, *g* = 9.81 m/s$^2$, during the quiescent time between two events. The acceleration spike of $\hbox {U}_2$ is well above 1.2 *g* or about 3 orders of magnitude higher than the background noise. The initial cylindrical sample shape is completely deformed in a fast liquefaction failure $\hbox {U}_3$ with sustainable vanishing effective stress (vertical red arrow in Fig. 1, red solid circle in Fig 2 and Supplementary Movie S2) and very large deformation triggered at only 212 kPa of all-round confining stress. It is then not possible to resume the compression test after this ultimate failure.

Each instability event can be characterised by three macroscopic parameters simultaneously with the presence of noticeable audible crackling noise: a sudden rise of solid fraction $\Delta \Phi$ indicating a compaction in volumetric strain $\Delta \varepsilon _{v}$, a contraction in axial strain $\Delta \varepsilon _{a}$, and a very fast reduction of $\sigma '$ due to spontaneous outburst of pore fluid pressure *U*, followed by a relatively slow recovery of $\sigma '$ in the dissipation of the excess pore fluid pressure. The internal stress $\sigma '$ of the saturated granular structure is greatly reduced by *U*, according to the Terzaghi effective stress principle, $\sigma '$ = $\sigma$ - *U*^18^. The last event $\hbox {U}_3$ has a vanishing internal stress accompanied by an exceptionally large incremental axial strain of 19.2% and a jump in solid fraction of 0.018 in one single step in less than 1 s, moving *I* toward a higher value of about 10$^{-3}$; hence the dynamic isotropic liquefaction for the slip phase^16^, crossing the jamming transition^21^ and the dynamic consolidation for the stick phase^19^. Note the consistent positive delay of pore pressure outburst for all instability events, indicating a surprising counter-intuitive coupled response with systematic constant time delay^22^ between the solid and liquid phases for a fully saturated two-phase mixture (Fig. 2). This unexpected pore fluid pressure outburst is fully assessed by two sets of static and dynamic pressure sensors at sample top and bottom^19^. The inconsistent time delay of $\Delta \varepsilon _{a}$ and $\Delta \varepsilon _{v}$ are probably due to the insufficient sensitivity of the LVDT displacement sensors to measure the fast and small changes. Future work using laser sensors inside the triaxial chamber will address this problem.

**Figure 3:**
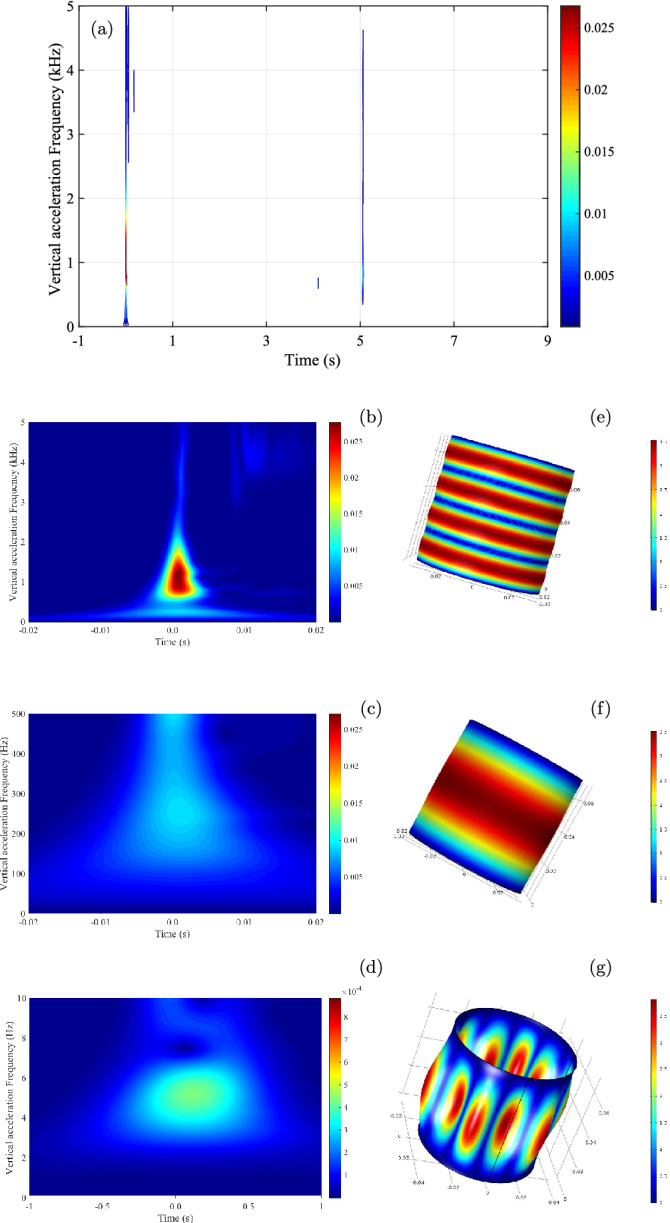
Spectrogram $|T_{\psi }(f,t-t_i)|^{2}$ using Continuous Cauchy Wavelet Transform^11^ on a segment of non-stationary signal of 10 s of vertical acceleration in isotropic liquefaction $U_{3}$ at 212 kPa (top). Details of spectrogram (left) and multiphysics numerical simulation^46^ (right) on 1175 Hz (top), 253 Hz of solid isotropic linear elastic medium (middle), 5 Hz of thin latex membrane (bottom).

With the Cauchy continuous wavelet transform (CCWT) and *n* = 100, we create the two-dimensional time-frequency spectrogram $|T_{\psi }(f,t-t_i)|^{2}$ to extract the instantaneous frequency of the extreme failure $U_{3}$ in isotropic compression in Fig. 3 for 10 s around the main event at $t_i$, and follow this frequency locally in time. The origin of time is shifted to the beginning of changes ($t = t_i$) in the vertical acceleration. The current time resolution is ± 0.1 ms. The prominent features concern the simultaneous and the short-lived excited frequencies during each isotropic slip. These frequencies over a broad range up to 5 kHz are visible as very thin colored vertical bands in Fig. 3a, meaning very short duration. The CCWT can produce an accurate estimate of the duration and the amplitude of each excited frequency. The largest frequency at 1175 Hz has a strongest magnitude of 27.7$\times 10^{-3}$ (a. u.) and smallest duration in less than 5 ms with slightly asymmetric shape centered on 0.8 ms in Fig. 3b. A close-up view reveals a faint additional frequency at 253 Hz with a magnitude of only 9.3$\times 10^{-3}$ in double duration in Fig. 3c and an even fainter low frequency at only 5 Hz in Fig. 3d with longer duration. These low-frequency vibrational modes happen almost simultaneously. We also note the high frequency above 4 kHz in Fig. 3b as in the persistent bright horizontal bands with a very low magnitude of about 3$\times 10^{-6}$ across a typical quiet segment of 1 s between two events in Fig. S2. Comparing to this quiescence, the extreme isotropic slip can produce acceleration spikes as much as 4 orders of magnitude higher.

With reference to the high-frequency mode of the quiet period, we consistently identify a broadening of low-frequency vibrational modes at times of local and especially global failure, as frequently observed in granular materials^9,10,23^. We have clear differences in vibrational modes between the pre-failure, the failure and post-failure segments of the spectrogram. This temporal heterogeneity can be used for characterizing and tracking the instability event. The dominant frequencies shown in Fig. 3 with a better temporal resolution for the event duration is reminiscent of the modograms created by the densities of vibrational modes that have been proposed in^9^ to follow the changing state of a granular system having a single layer of large grains in an annular shear cell^24^. However, its constant frequency within fixed temporal window has the disadvantage of poorly time localization.

Not all low-frequency vibrational modes are detected simultaneously by our three complementary sensors in Fig. S3. The vertical acceleration has the most accurate time-frequency localisation, probably due to the direct measurement on the sample top cap. The indirect lateral measurements using high-performance portable acoustic sensors, located outside the triaxial chamber, may contribute to less precise results. All the vertical and lateral short-lived events rise instantaneously, peak simultaneously within 5 ms and last less than 20 ms. These different directional acoustic transmissions can be attributed to the spatial heterogeneities of the force chains ruptures, of the contact network in granular materials^25–27^ and to the initial anisotropic structure of our granular assemblies created by the moist-tamping method^16^.

Table 1 gives separately the four vibrational modes using continuous wavelet technique for a set of more than 30 collapse and liquefaction experiments. The main average dominant frequency at liquefaction (341 Hz) is greater than that of collapse (311 Hz) since the solid fraction of the liquefaction event is more compact. This technique can characterize the changes in frequency at slip time and identify the complementary time at peak amplitude and the very short total duration of the event in less than 10 ms in Fig. S4. The relative large standard deviation in this table might be linked to the wide range of uncontrolled triggering conditions (effective stress from 20 up to 500 kPa, solid fraction from random loose packing up to random dense packing) at the time of instability event.

To the best of our knowledge, these laboratory dynamic instabilities are a new kind of laboratory earthquake and completely unknown to theoretical scientists, despite the development of numerous advanced constitutive models for geomaterials in the last decades^28–34^, even to numerical ones with the recent explosion of DEM (discrete element modelling) simulations on various particle shapes^35–43^. The observational behaviours, especially the sample destruction under usual laboratory loading conditions, i.e. low confining stress ($\leqslant$ 1 MPa), low stress rate ($\leqslant$ 0.1 kPa/s) and ambient temperature ($\leqslant$ 25 $^{\circ }$C), cannot be explained by various evolutional laws of rate-and-state-frictional behaviour^5–7^, and by recent theoretical frameworks of frictional dynamic^44,45^. Furthermore, they never happen for natural granular materials (i.e. sands) and according to classical granular physics, the extreme isotropic liquefaction failure in saturated isotropic granular assembly is conceptually impossible^16^.

However, the physics of the observational vibrational modes can be explored using the FEM numerical simulation with acoustic modal analyses of Comsol Multiphysics^46^ on a short full cylinder of homogeneous and simple plastic material consisting of isotropic linear elastic behaviour with Mohr-Coulomb plastic criterion. For cohesionless granular soil with constant frictional angle, density and Poisson modulus, simple trial and error procedures on the Young modulus *E* permit to fit the first known measured modal frequency of 253 Hz for the most destructive event $U_{3}$ with *E* = 11.74 MPa. Consequently, the mechanical characteristics of a granular system, especially *E*, is encoded indirectly within the dominant frequency detected by CWT.

This axial vibration frequency is very close to the musical note $\hbox {E}_{4}$ above middle $\hbox {C}_{4}$, in the audible range; and can give a plausible explanation on the origin of the systematic hearing crackling noise of each event. Since each vibrational mode is associated with a specific frequency, the frequency at 1175 Hz corresponds to the 4th vibration mode visible as four horizontal red bands in Fig. 3e, while the first mode at 253 Hz is still active in Fig. 3f as a single large red band. This is the characteristic sound of the granular instability. The lowest and faintest identified frequency at 5 Hz can be attributed to the first vibrational mode of an open-ended latex membrane enclosing the granular sample in Fig. 3g. The computed solutions to the Bessel differential equations for these simple cases can be found in^47^.

The audible frictional sound of triaxial compression stick-slip can also be partially explained by the vibration of two adjoining grains associated with the sudden breakage of the force chains^48,49^.

**Table 1:** Identification of vibrational modes of isotropic collapse and liquefaction instabilities by CCWT and the associated average frequency.

Mode	Collapse	Liquefaction
n	Hz	Hz
1	311.4 ± 74.4	341.4 ± 59.6
2	667.7 ± 56.6	653.2 ± 74.2
3	877.0 ± 74.2	884.5 ± 69.8
4	1322.2 ± 144.6	1268.4 ± 132.2

**Table 2:** Parameters of isotropic collapses $U_{1}$, $U_{2}$ and liquefaction $U_{3}$ in isotropic compression from 20 to 212 kPa of confining pressure.

	$U_{1}$	$U_{2}$	$U_{3}$
	Collapse	Collapse	Liquefaction
Confining stress $\sigma '_{3c}$ (kPa)	57	92	212
Dominant frequency (Hz)	154	204	253 (+1175)
Amplitude 10$^{-3}$ (a.u.)	2.84	3.94	9.30 (27.66)
Young’s modulus *E* (MPa)	4.36	7.64	11.74 (253.1)

Table 2 and Fig. S5 give some characteristics and the estimated Young modulus *E* for all events and show a loss of rigidity with decreasing frequency, cell pressure and even solid fraction, as for low-frequency vibrational modes in^10,50–52^. Numerically, the dominant frequency follows *E* as a power law with an exponent of 0.5 (Fig. S6). The estimated low values of *E* is probably due to simple linear isotropic elastic materials and to small internal effective stress near the jamming transition. However, intuitively, a null *E* is expected at liquefaction with vanishing effective stress while increasing strain in Fig. 2, as also suggested by a continuously decreasing shear wave velocity $V_p$ with decreasing normal stress in unloading^53^.

**Figure 4:**
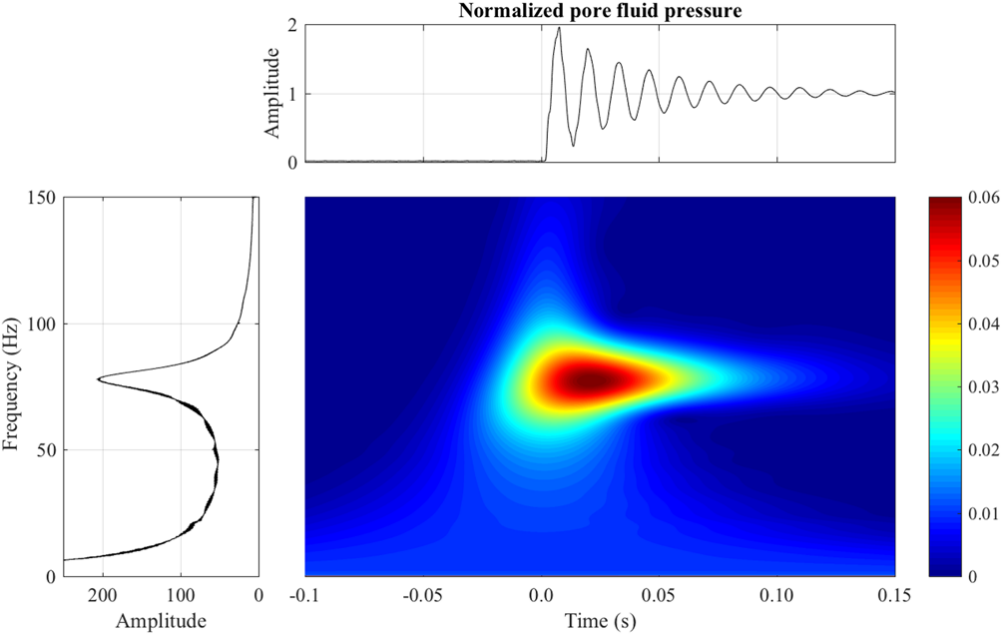
Continuous Cauchy wavelet transformation of the excess pore fluid pressure or intergranular stress with full destruction of saturated granular assembly (isotropic liquefaction $U_{3}$) at 212 kPa of confining pressure.

## Discussions

The effective or internal stress $\sigma '$ on a saturated two-phase granular structure can be estimated using Terzaghi effective stress principle^18^. With constant radial stress $\sigma$ inside the triaxial chamber and constant static back pressure $U_0$, the double verified measurement of pore fluid pressure *U* gives access to $\sigma '$ and Fig. 4 shows the spectrogram of $\sigma '$ or *U* happening within 0.25 s. The systematic delay of *U* (vertical black dashed line), of about 2 ms behind the changes of vertical acceleration in Fig. 2 indicates a surprising coupling response with systematic constant time delay^22^ between the solid (granular skeleton) vibrated at 1175 Hz and the pore fluid (de-aired and distilled water) at only 77 Hz. Furthermore, the duration of pore fluid regular vibration, of about 0.2 s, is more than one order of magnitude longer than all others. This special coupling response and the additional vibrational features cannot be explained with current knowledge of granular physics. However, while not being a definitive causative mechanism of the observed destructive event, this pore fluid pressure outburst is a distinctive signature to the vanishing intergranular stress and a requisite condition for liquefaction^16,19^. The magnitude and the duration of this pore pressure surge play a particularly significant role in the destruction of the granular structure. This perplexing vibration of the intergranular stress at constant cell pressure is another still unexplained signature of the instability in isotropic compression.

**Figure 5:**
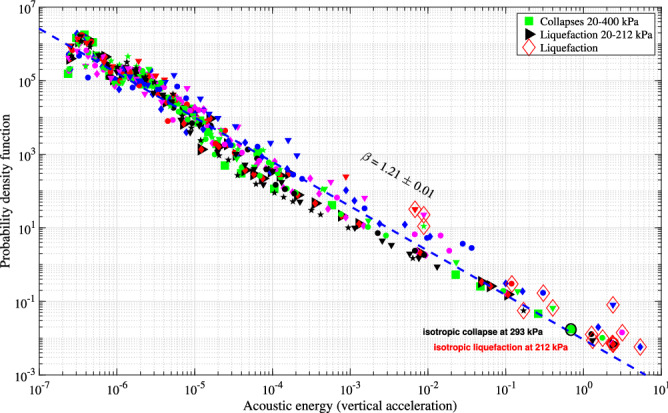
Probability density distribution of acoustic energy $E_a$ of the vertical top cap acceleration for isotropic collapses (solid symbols) and liquefactions (large hollow red diamonds) events. The blue dashed line represents the power-law behaviour, $P(E_{a}) \sim E_{a}^{-\beta }$, $\beta$ = 1.21 ± 0.01, R$^2$ = 0.967. The symbol ± stands for 95% confidence interval.

Various plausible explanations have been proposed, from systematic errors to the presence of grain doublets and to new granular physics^54^; and improved measurements of this paper have not resolved the mystery of granular dynamic instability. The interpretation of the isotropic liquefaction event still remains a matter of debate; as even the simple time process of pore-water pressure dissipation in phase *III*^19^. The systematic changes in top cap acceleration using high-temporal-resolution measurements suggest a fast underlying modification of the granular microstructure favoured by a very inhomogeneous environment, creating a sharp increase of the pore water pressure on a timescale of milliseconds in a simple two-phase (solid and liquid) granular assembly, and followed by a dynamic consolidation towards a more compact structural rearrangement, as in stick-slip experiments in triaxial compression with shear stress^22^.

The simple isotropic compression can generate a broad energy distribution, spanning over 7 decades. Figure 5 presents the probability density function of the acoustic energy measured by the vertical top cap acceleration $E_{a}$ for a set of new 16 liquefactions (hollow red diamonds) out of more than 110 experiments^55^, with the addition of one representative compression test up to 400 kPa having no liquefaction event. This new set showed that the earlier prompt detection of granular instabilities^16^ was not a fortunate accident. The energy-frequency distribution, binned logarithmically, spreads over almost eight decades and follows a single power-law with no low or high energy cutoff $P(E_{a}) \sim E_{a}^{-\beta }$, similar to the statistical seismic power-law for natural earthquakes (Gutenberg–Richter^56^). The measured slope of the power law behaviour (dashed blue line) has high correlation coefficient across 17 experiments totalling more than 3$\times 10^{4}$ events, $\beta$ = 1.21 ± 0.01 (R$^2$ = 0.967). This power-exponent encompassing largest isotropic labquakes is lower than that of worldwide earthquakes with $\beta$ = 5/3 = 1.67^57^, meaning a significant increase of the occurrence of high energetic labquakes, up to one order of magnitude greater than earthquakes, for a given energy above 10$^{3}$. However, this low value might be a result of the small laboratory sample size. The very simple isotropic compression experiments extend the traditional statistical power-law relation in seismicity on the upper end with extreme liquefaction failure and very large deformation under uncontrolled triggering stress.

Intuitively, the liquefaction failure at only 212 kPa with largest incremental axial strain and vanishing effective stress is amongst the most destructive one, in terms of irreversible damage with substantial structural rearrangement, and the most energised event. The acoustic energy needed to liquefy at 212 kPa (red hollow circle) is greater than that for collapse at 293 kPa (black hollow circle). However, the ability of isotropic liquefaction failure to occur anywhere in the studied range from 20 up to 500 kPa^16^ deepens the mysteries on the causative mechanisms of these granular instabilities. Figure S7 gives a power-law with high correlation (R$^2$ = 0.922) between the acoustic energy and the isotropic triggering stress for the final liquefaction failure. Higher confining pressure can generate liquefaction event with larger acoustic energy. This correlation also suggests the possibility of liquefaction well below 20 kPa, associated with very low acoustic energy, as happened in loose and saturated shallow granular soils^58^. Both the lower and upper ends of this power-law correlation are not known and are the subject of current research.

The empirical Gutenberg–Richter’s law for natural earthquakes can be partially generated by DEM simulations of dense granular materials under uniaxial compression^38,59^. However, these numerical simulations show a repetitive small stick-slip behaviour and follow a power-law distribution with a stretched exponential cutoff for less than 3 decades. By contrast, our experiments in isotropic compression offer very large liquefaction failure and a power law with no low energy cutoff over almost eight decades.

We have also identified three basic ingredients for the appearance conditions of these dynamic instabilities: two for the isotropic collapse (an initial density from very loose to medium dense and an initial structural anisotropic state) and one additional for the isotropic liquefaction (the duration of the stabilised excess pore pressure in phase *II* should be greater than 1 s)^16,17^. Furthermore, stick-slip frictional failures happen regardless of the saturation (dry, water-wet and water-saturated) of the model granular assembly, the initial effective confining pressure up to 500 kPa^60^, the sample height-to-diameter ratio^61^ and even in mixtures of sand and glass beads^62^. The reproducibility, in the sens of exact replicability of the results, cannot be achieved due to the uncontrolled triggering stress of the instability event; however, it is achieved in the sens of repeatability of the instabilities (Fig. S7).

## Conclusions

We have proposed a non-invasive method through passive recording of vibrations and acoustic emissions for assessing the evolving stress state of a granular system during the stick-slip-like event in simple isotropic compression using the Cauchy continuous wavelet transformation. From numerical vibro-acoustic simulations of simple plastic granular materials, the identified short-lived low-frequency vibrational modes with better temporal resolution can be partly interpreted as the axial vibration of a thick cylinder of granular materials in a particle frequency in the hearing range. Nevertheless, what are the physical causes of these unusual granular instability events in simple isotropic compression, especially the largest laboratory quakes to date? Many details of the main triggering mechanisms still remain elusive to us.

The proposed non-invasive acoustical method can be used as an alternative method in early detecting imminent failure, as in the density of vibrational modes^9,10^, the sudden change in the statistical seismic power-law exponent^63^, the acoustic emission rate^64^, the acoustic energy release^65^ for citing a few. In our case, we use a Cauchy continuous wavelet transform with simple measurements of vertical acceleration or of excess pore fluid pressure to have only 1 s warning in our experiments.

We were able to establish that there is room to improve on existing theories, and discover a better, more accurate phenomenological constitutive or numerical model for granular physics, enhanced with a new instability phenomenon for rounded particles, similar to the stick-slip phenomenon. Based on our experimental observations, it seems that no known physics can account for these perplexing instability events and probably some unknown fundamentals are still missing in current theoretical and numerical models. Current theoretical models cannot resolve even the simple relaxation time or the permeability coefficient in the dissipation phase *III* of the two-phase mixture in Fig. 2^19^. The ability to predict these dynamic instabilities is a very difficult research challenge for modern discrete element modelling technique, primarily performed on spherical particles, especially with a full solid-liquid coupling formulation.

The broad energy distribution of recoverable isotropic collapses and ultimate liquefaction failure of our simple experimental system can extend the usual statistical Gutenberg–Richter correlations for hypothetical largest earthquakes based on the mechanisms of stick-slip frictional instability. The more we know about the extreme laboratory quakes, especially in isotropic compression, the more we are one step closer to improving our understandings of the natural catastrophic earthquakes through the extended statistical power-law on the energy-frequency distribution of very large isotropic instability events. The next experimental works on triaxial compression stick-slip with the presence of additional shear stress should confirm these previous findings.

## Supplementary Information

Supplementary Information.

Supplementary Movie S2.

## Data Availability

The datasets used and/or analysed during the current study are available from the corresponding author on reasonable request.
