# Supplementary material for: An acoustic signature of extreme failure on model granular materials
Source: Sci Rep. 2022 Oct 31;12:18304. doi: 10.1038/s41598-022-20231-6 (PMC9622919; doi:10.1038/s41598-022-20231-6)
Supplement: Supplementary file 1 — Supplementary Information. [file 41598_2022_20231_MOESM1_ESM.pdf]

An acoustic signature of extreme failure on model granular materials
Supplementary Materials

T.T. Nguyên,[1] T. Doanh,[1,*] A. Le Bot,[2] and D. Dalmas[2]

[1]*Ecole Nationale des Travaux Publics de l'Etat. LTDS (UMR 5513). Vaulx en Velin. France*
[2]*Ecole Centrale de Lyon. LTDS (UMR 5513). Ecully. France*
[*] *thiep.doanh@entpe.fr*

A. Experimental details

For this study, the granular materials were created from industrial soda lime glass beads "Sil-glass" commercialized by CVP, Linselles, France[S1]. The quasi-perfect spherical glass beads SLG 6-8 have a mean grain diameter, $d = D_{50}$ = 0.723 mm, a mean surface roughness Sa, and a arithmetic mean height, of only 36 nm.

Short cylindrical granular sample, H_0 = 70 mm in height and D_0 = 70 mm in diameter, is fabricated using a modified moist tamping and under compaction method [S2, S3], mixed with 2% of distilled water by weight, then placed in the triaxial mould in five layers, compacted up to prescribed thickness inside an open ended cylindrical latex membrane of 0.3 mm of thickness.

To ensure a full saturated state, we use CO_2 method [S4] under very low gradient of only 0.2 kPa, followed by flushing de-aired and distilled water and application of constant back-pressure U_0 of 200 kPa to obtain a high Skempton's coefficient B = $\Delta U/\Delta\sigma \geqslant 0.95$ [S5].

For the simple isotropic compression under stress-controlled mode, we applied slowly compressed air to a desired cell pressure $\sigma'_r = \sigma'_3$, the radial (minor principal) stress. The sample is not kinematically constrained in the vertical direction since the vertical loading ram is not physically connected to the sample top cap. Consequently, the sample top cap is free to move vertically when sheared quasi-statically either in stress (isotropic compression) or strain-controlled (triaxial compression) loadings.

Comparing to past studies [S6, S7], the new acoustic measurements consist of one tiny piezoelectric accelerometer with resolution of 350 μg within 10 kHz (PCB 607A11) to measure the instantaneous vertical acceleration of the sample top cap inside the triaxial chamber; plus one complementary non-contact portable laser vibrometer, Ometron OH-100-D, and one free-field prepolarized microphone, BSWA MPA408 with ICCP preamplifier MA408, to measure the local lateral surface vibration at mid-height of the sample latex membrane and the radial sound pressure at the sample bottom outside the triaxial chamber.

We measure the pore water pressure at the sample top and bottom with two set of complementary pressure sensors: one static pore-water pressure U (Sedeme, MD20) with an unknown resonant frequency, complemented by one dynamic piezoelectric pore pressure sensor (PCB S112A21) with a high resonant frequency (250 kHz) to verify the fast rise-time and the unexpected oscillating nature of U.

We use two dynamic data acquisition boards NI 4472B of National Instruments to measure concurrently and synchronously all static and dynamic sensors at minimum 10 kHz to follow the fast dynamics of the isotropic collapse and liquefaction in detail.

The triaxial apparatus is located in a separated room and isolated from external building vibration by passive isolation systems.

B. Isotropic collapse and liquefaction dynamics

Fig. 2 gives a typical temporal evolution of isotropic collapse U_2 (blue) and liquefaction U_3 (red) in isotropic drained compression. A semilogarithmic time scale is used to follow the temporal behavior over fiver orders of magnitude with focusing on the short time scale below 10 ms. The time scale is shifted to the beginning of the changes in acceleration data ± 0.1 ms, which is the current time resolution. The first peak in the acceleration data, occurring at 0.8 ms and up to - 1.5 g for the liquefaction event, is representative of a sudden fall of the sample top cap. This first acceleration peak always precedes that of the pore pressure by 2.0 ms. After this time delay, the pore fluid pressure changes rapidly from the imposed constant back pressure U_0. Three phases characterize this sudden change [S6]. First, a transient phase I in which the pore pressure rises instantaneously from the constant back pressure U_0 and fluctuates toward a stabilised value U_{stable} within 200 ms. Then an intermediate phase II at constant U_{stable} before a slow dissipation phase III toward U_0. The duration of phase II, longer than one second, is a characteristic for the specific

liquefaction case, as U_3. To compare different stick-slip like motions, the excess pore pressure $U - U_0$ is normalised by the stabilised value $(U - U_0)_{stable}$.

In saturated granular mechanics, liquefaction is associated with a vanishing effective stress, $\sigma' = \sigma$ - U = 0 [S8]; it means the total stress σ is equal to the pore fluid pressure U. For the liquefaction U_3 in Fig.1 in linear scale, the vertical red arrow indicates a null effective isotropic stress $\sigma' = 0$ during a lengthy evolution of the solid fraction. For the same liquefaction U_3 in Fig.2 in logarithmic time scale, the normalized excess pore pressure, $(U - U_0)/(\sigma - U_0)$ = $\Delta U/\Delta\sigma = U/\sigma$ (for U_0 = 0 kPa) = 1 (red solid circle); thus $\sigma' = 0$ and the liquefaction failure associated with a very large axial strain of 19.2% and a destruction of the sample shape.

Furthermore, for the liquefaction failure U_3 in isotropic compression under constant stress rate in Fig.1, we observe an unexpected jump in solid fraction of 0.018 (vertical red arrow at constant stress); and in the temporal complementary Fig.2, an exceptionally large incremental axial strain of 19.2% in one single step in less than one second. This duration of the liquefaction event of one second (or 0.037% of the total experimental duration) can be qualified as spontaneous regarding the usual time scale of the isotropic compression of about 45 mins to increase the effective cell pressure from 20 to 212 kPa.

The spontaneity of the failure event can be seen in the changes of the vertical acceleration in less than 0.01 seconds in Fig.2 (0.00037% of the total experimental duration), or in the changes of the pore fluid pressure in less than 0.1 seconds (0.0037% of the total experimental duration). Normally, these short-lived changes should not happen in a quasi-static regime under low dimensionless inertial number $I \approx 10^{-6}$ [S9] in drained loading condition (constant pore fluid pressure).

Liquefaction in isotropic compression occurs under specific conditions [S6]: (1) the duration of phase II longer than one second, (2) the normalised excess pore pressure equals unity or the effective stress approaching zero value, and (3) an initially loose granular assembly above a threshold void ratio. If the duration of phase II is less than one second, the instability event is termed as isotropic collapse, and the compression experiment can resume without the destruction of the granular structure.

The incremental axial strain $\Delta\varepsilon_1$ and the incremental volumetric strain $\Delta\varepsilon_v$ show a dynamic axial slip in less than one second; and the the short-lived evolution of the pore fluid pressure happens essentially in undrained condition (no pore fluid flow).

Fig. S1 gives the typical temporal evolution of the vertical top cap acceleration of the second event U_2 in the normal linear scale of 200 seconds from arbitrary beginning time, complementary to the logarithmic scale in Fig. ??. It shows a sharp spike of acceleration up to 1.5 g, the gravity acceleration g = 9.81 m/s^2, at t = 11.571 seconds with practically no precursors (foreshocks) and very small aftershocks, comparing to real earthquakes. The top inset shows the close-up view of the main short-lived event U_2 of large magnitude near 1.5 g, lasting less than 5 ms; and the bottom inset the quasi-static regime of isotropic compression below 0.002 g under low stress rate ($\leqslant$ 0.1 kPa/s) in a segment of 20 seconds between two main events U_2 and U_3. The typical background noise level of acceleration signals is of about 29 dB. The cell pressure is constant at 295 kPa and the prescribed constant back pressure U_0 at 200 kPa in this segment.

The cylindrical sample shape is destroyed in a rare isotropic liquefaction failure U_3, as shown in the supplemental movie M1a, acquired with fast camera at 3 000 fps (frames per second) and viewed at 30 fps. This movie is the first ever recorded isotropic liquefaction on fast camera.

C. Cauchy continuous wavelet transform

Fig. S2 (top) shows a typical spectrogram using a continuous Cauchy wavelet of 100th order of a quiet segment of one second of the vertical top cap acceleration preceding the second isotropic collapse U_2, showing the nearly continuous frequency as a large horizontal red and blue band centered above $\sim$ 4 kHz. The close-up view in Fig. S2 (bottom) reveals the discrete details in a segment of 0.02 seconds (from 0.10 to 0.12 seconds) with a local short-lived power peak at 4.4 kHz and 0.115 seconds with a faint magnitude of only 6×10^{-6} (au), about 4 orders of magnitude lower than that of typical isotropic collapse event. We can follow closely the temporal evolution of this local peak in the time-frequency analysis.

Fig. S3 and table I give the Cauchy spectrogram and the vibrational characteristics by three complementary sensors in two perpendicular directions of the extreme failure event U_3 in a segment of 0.04 seconds, centred on the abrupt change of the vertical top cap acceleration G (top), the radial vibration with non-contact portable laser vibrometer V (middle) and the lateral sound pressure with free-field prepolarized microphone M (bottom). Each figure shows the temporal evolution of the time series in linear scale on top subfigure, its spectrum on the right subfigure and the spectrogram on the main panel. The vertical acceleration has the largest magnitude and the smallest duration, and two order of magnitude greater than that of lateral sound pressure.

Fig. S4 indicates the time at peak amplitude and the very short total duration below 0.01 seconds for all isotropic

TABLE I. CCWT parameters of spontaneous liquefaction in isotropic compression at 212 kPa, from complementary acoustic measurements in vertical and horizontal directions.

	Vertical acceleration	Radial vibration	Lateral sound pressure
Frequency (Hz)	1175	690	1400
Amplitude 10^{-3} (a.u.)	27.664	0.544	0.115
Peak time (ms)	0.800	5.100	2.400

events in a series of 17 experiments up to 500 kPa of confining pressure: collapse (a) and liquefaction (b), plus the liquefaction from the first stick-slip in triaxial compression (solid triangle) and Fig. S5 the associated dominant frequencies, especially the first mode around 300 Hz, function of stabilized excess pore pressure.

D. Multiphysics numerical simulations

We use Comsol Multiphysics [S10] with acoustic and geomechanic modules to explore the observed vibrational modes on a short full cylinder of homogeneous and simple plastic material consisting of isotropic linear elastic behaviour (Young modulus E, Poisson modulus ν and density ρ) with Mohr-Coulomb plastic criterion (cohesion c and frictional angle ψ). For simplicity, the two end conditions are clamped (no displacement for top and bottom caps), and free lateral surfaces are allowed. The finite element mesh of the thick cylinder has 14 000 tetrahedral elements; and that of the thin hollow cylindrical latex membrane enclosing the granular cylinder 15 000 tetrahedral elements.

With fixed $\rho = 1.428$ kg/m^3, $\nu = 0.25$ and cohesionless granular soil $c = 0$ kPa, $\psi = 31^o$, the first known measured modal frequency of 253 Hz for the liquefaction event U_3 at 212 kPa of confining stress is fitted using simple trial and error procedures to identify the Young modulus $E = 11.74$ MPa. It seems that the granular cylinder and the membrane are decoupled in numerical experiments since further simulations with a thin latex membrane enveloping a thick granular cylinder do not reveal the lower modes below 20 Hz.

In Fig. S6 on double logarithmic axis, the frequency of the vibrational modes and the estimated bulk modulus has a perfect power law relationship with an exponent of 0.5.

E. Acoustic energy

We use the temporal series of vertical top cap acceleration, as in Fig. 2, to determine the beginning of each instability event (isotropic collapse or liquefaction). The event signal was detrended, smoothed with Savitzky-Golay filter of 7th order to ease the event peak and duration detection. The local peaks are detected as the local maxima larger than the two neighboring data using the derivative based finding algorithm in ”findpeaks” function of Matlab [S11]. Then the beginning $t_{b(i)}$ and the end $t_{e(i)}$ of each event are indicated by a sudden change of the first derivative of the smoothed temporal signal indicating the stabilisation backward and forward below a noise threshold from the local peak. The threshold for detecting the vertical acceleration is set to 0.005 g, larger than the actual noise level in Fig. S1. For isotropic liquefaction failure, the peak signal can be as large as 16 g.

The acoustic energy E_a associated with the temporal series of the vertical top cap acceleration $G(t)$ is estimated by the squared maximal value of $G(t)$ with arbitrary unit. Regardless of the isotropic confining stress from 50 to 500 kPa, and of the uncontrolled triggering stress of the liquefaction failure in this stress range, the acoustic energy and the isotropic triggering stress, even uncontrolled, for the final liquefaction failure follow a highly correlated power-law ($R^2 = 0.922$) in Fig. S7 on double logarithmic axis.

The acoustic energy E_{ai} can also be estimated by the integral of $G(t)^2$ over the event duration from the beginning $t_{b(i)}$ to the end $t_{e(i)}$ of the stick-slip event (equation S1). In our case, E_a is proportional to E_{ai}.

$$E_{integration} = \int_{t_{b(i)}}^{t_{e(i)}} G(t)^2 dt \tag{S1}$$

The probability distribution of E_a is estimated on logarithmic bins with 10 bins par decade between the minimum and maximum of measured energy; and the frequency distribution is normalised by the bin width to give the probability density function (PDF). The power exponent is obtained using a nonlinear regression model ”fitnlm” of Matlab based on iterative least squares Levenberg-Marquardt method.

F. Alternative analysis for the probability density function of acoustic energy

The probability density function of the acoustic energy measured by the vertical top cap acceleration E_a can be evaluated in a double power-law with a crossover energy $E_c \approx 10^{-5}$ (a.u.) in Fig. S8. Below E_c, the energy distribution of small events yields a power exponent $\beta_1 = 1.11 \pm 0.04$ (mean ± deviation, $R^2 = 0.779$) over only two decades, and another power exponent $\beta_2 = 1.08 \pm 0.01$, $R^2 = 0.951$ beyond E_c for nearly six decades. The smaller second power exponent β_2 of the double power-law, 1.08 instead of 1.21, seems to better suit the liquefaction data of large acoustic energy than the simple power-law. However, the relatively low correlation coefficient for β_1 makes this alternate analysis less preferable than the single power-law in the main text. Consequently, additional experimental works are needed to sort out the best analysis.

It seems that the lack of low energy cutoff in Fig. 5 is not a consequence of our experimental setup. The sensitivity of our accelerometer can measure very light energetic events, down to 10^{-8}. Fig. S9 shows, as an example, the presence of a low energy cutoff $E_{min} = 2{*}10^{-6}$ (a.u.) for water-wet model granular assembly in stick-slip experiment of triaxial compression at 500 kPa of confining pressure.

[S1] Www.Cvp-abrasif-broyage.com.
[S2] Bjerrum, L., Krimgstad, S., Kummeneje, O., in *Proc. 5th Int. Conf. Soil. Mech. Found. Engrg.*, Vol. 1 (1961) pp. 29–37.
[S3] Ladd, R.S., Geotechnical Testing Journal **1**, 16 (1978).
[S4] Lade, P.V., Duncan, J.M., J. Soil Mech. and Found, ASCE **99**, 793 (1973).
[S5] Skempton, A. W., Taylor, R.N., Géotechnique **4**, 143 (1954).
[S6] Doanh, T., Le Bot, A., Abdelmoula, N., Hans, S., Boutin, C., Europhys. Lett. **108**, 24004 (2014).
[S7] Doanh, T., Abdelmoula, N., Nguyên, T.T.T., Hans, S., Boutin, C., Le Bot, A., Granular Matter **18**, 67 (2016).
[S8] Terzaghi, K., Peck, R.P., Mesri, G, *Soil Mechanics in Engineering Practice, 3rd Edition* (John Wiley, 1996).
[S9] Andreotti, B., Forterre, Y., Pouliquen, O., *Granular Media: Between Fluid and Solid* (Cambridge University Press, 2013).
[S10] COMSOL Multiphysics v.5.0, COMSOL, Inc. (2014).
[S11] MathWorks v.2014a, The MathWorks, Inc. (2014).

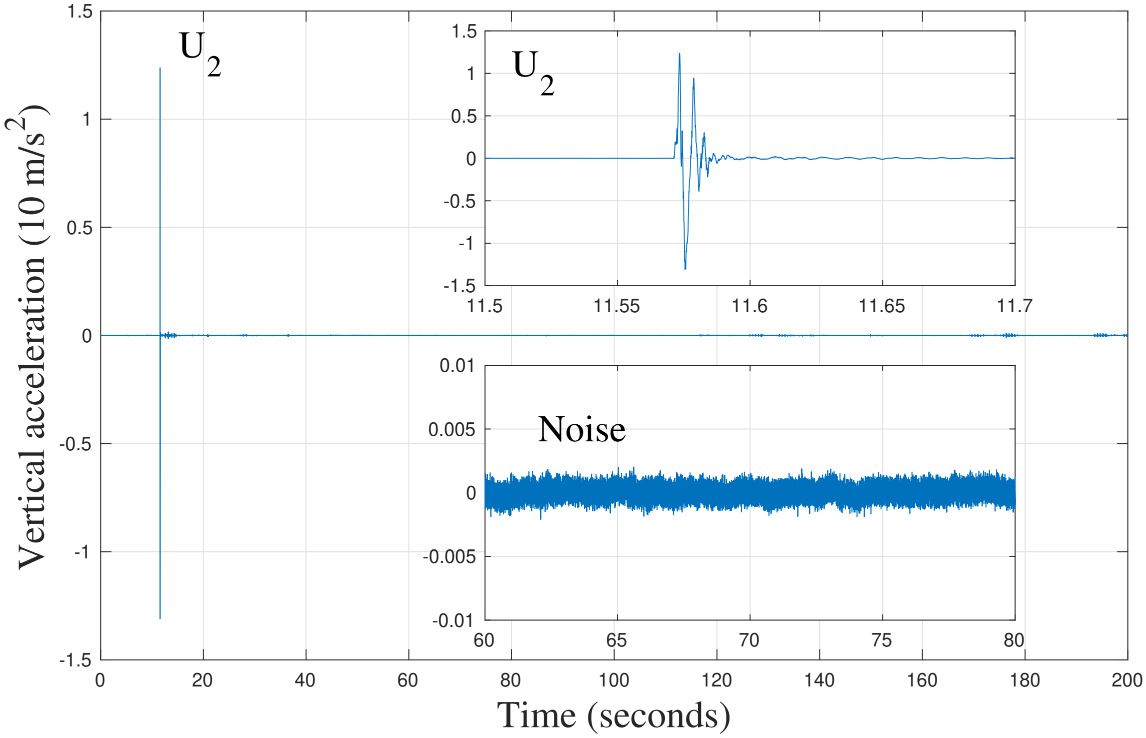

FIG. S1. Temporal evolution of the vertical top cap acceleration of the second instability event U_2 in a segment of 200 seconds with two close-up views on the main event and on a short segment of 20 seconds of quiet time.

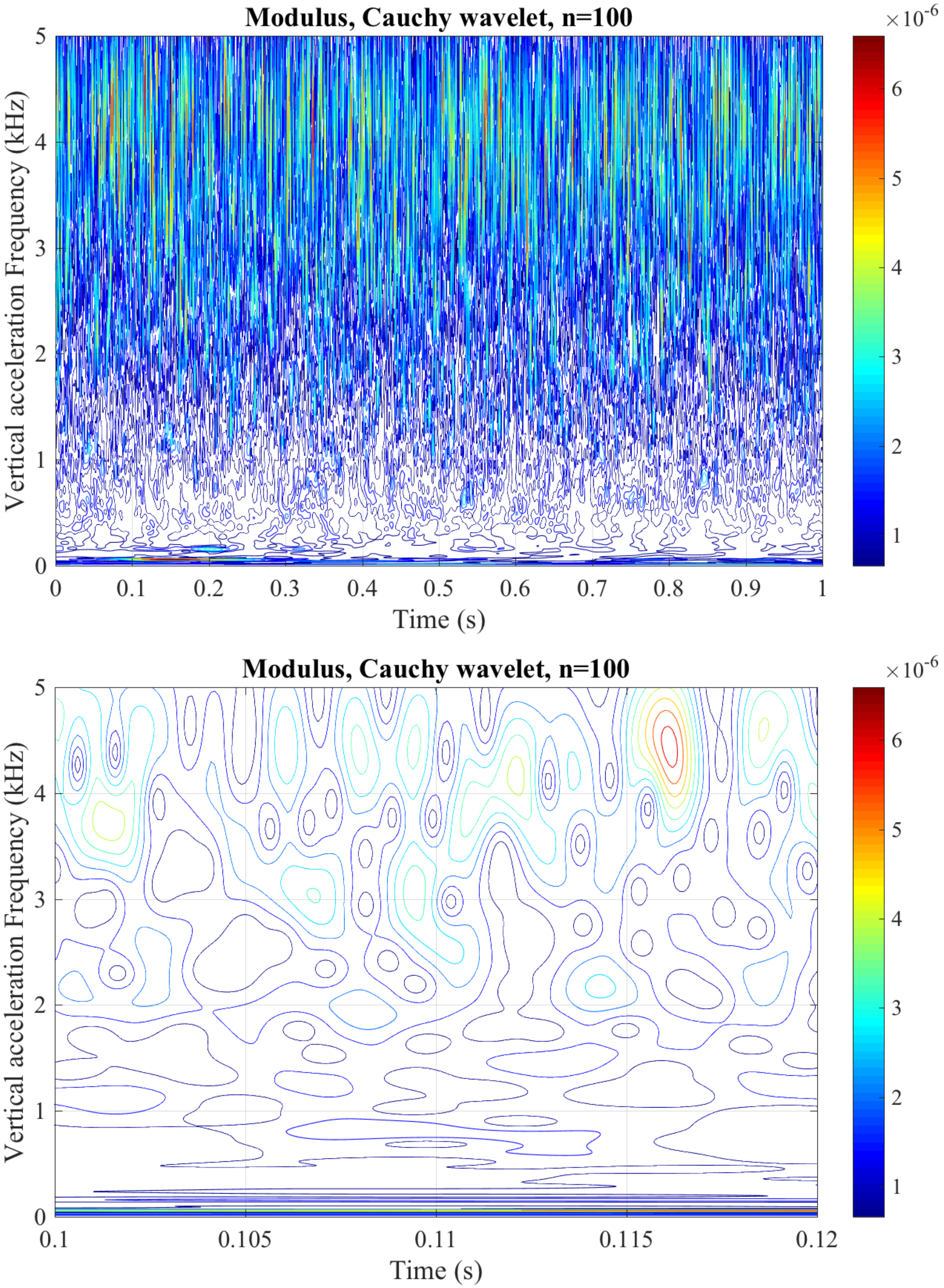

FIG. S2. Typical spectrogram using continuous Cauchy wavelet of a quiet segment of one second preceding the second isotropic collapse U_2 (top). Close-up view for a segment of 0.02 seconds starting from 0.1 seconds showing a string of nearly continuous vibrations above 4 kHz (bottom).

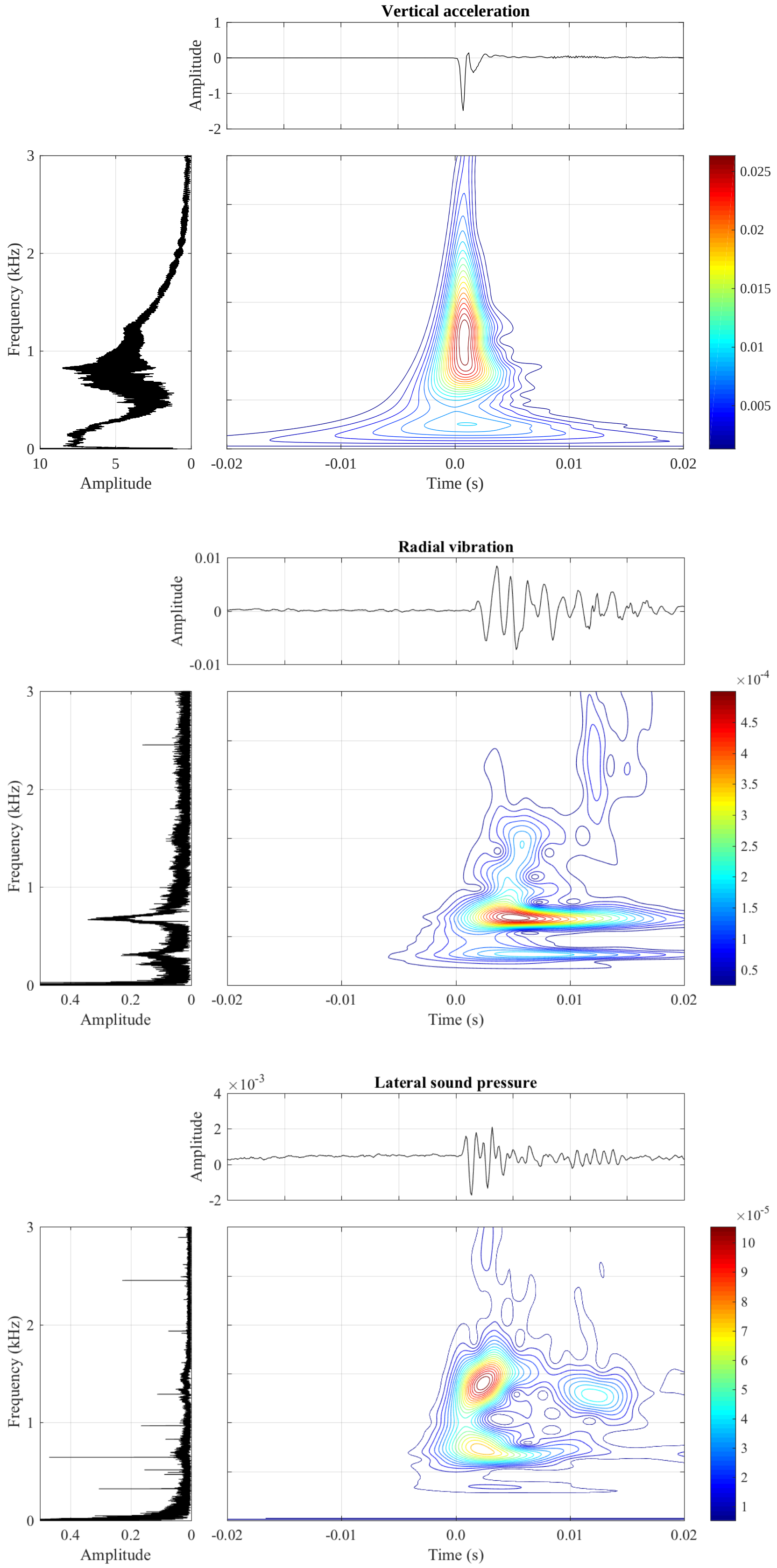

FIG. S3. Continuous Cauchy Wavelet Transformation of full destruction of granular assembly (isotropic liquefaction) at 212 kPa of vertical top cap acceleration (top), radial vibration (middle) and lateral sound pressure (bottom).

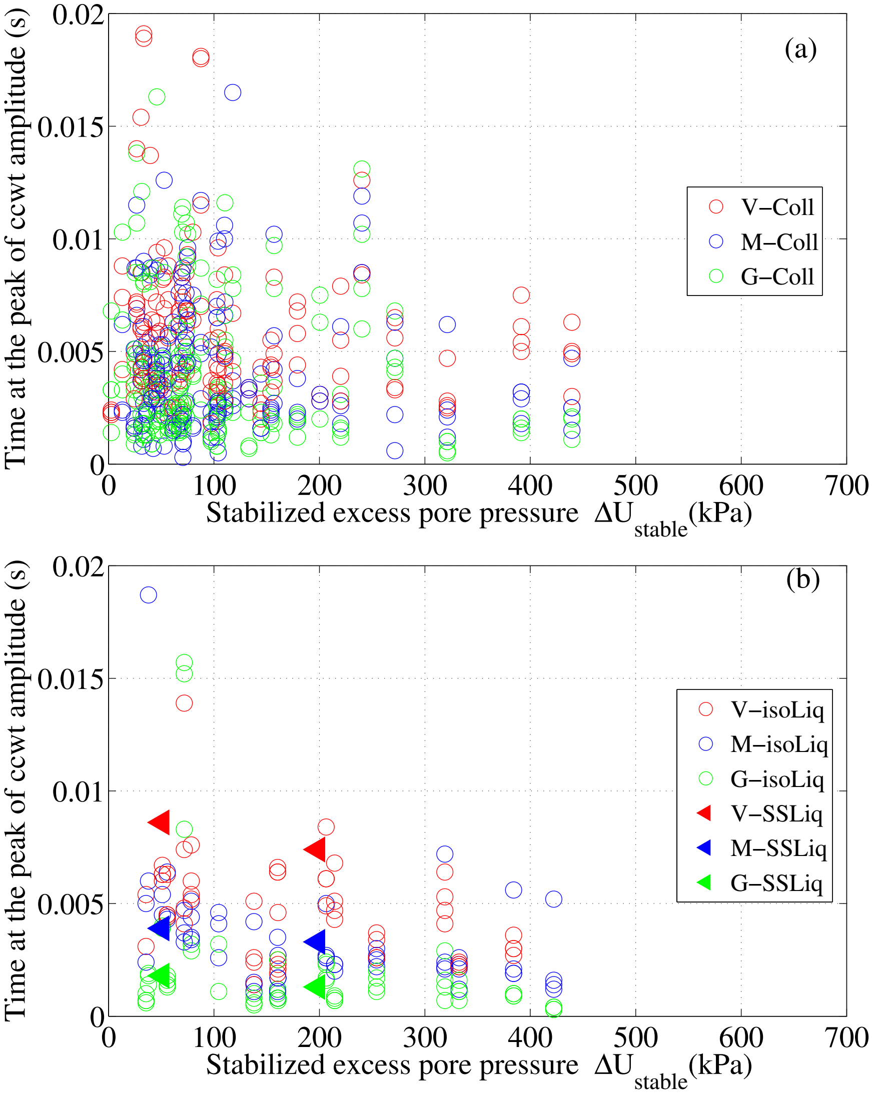

FIG. S4. Time at the peak of CCWT amplitude of collapse (a) and liquefaction (b) from vertical top cap acceleration G, radial vibration using non-contact laser vibrometer V and lateral sound pressure by microphone M. SSLiq means liquefaction from drained stick-slip events of triaxial compression experiment.

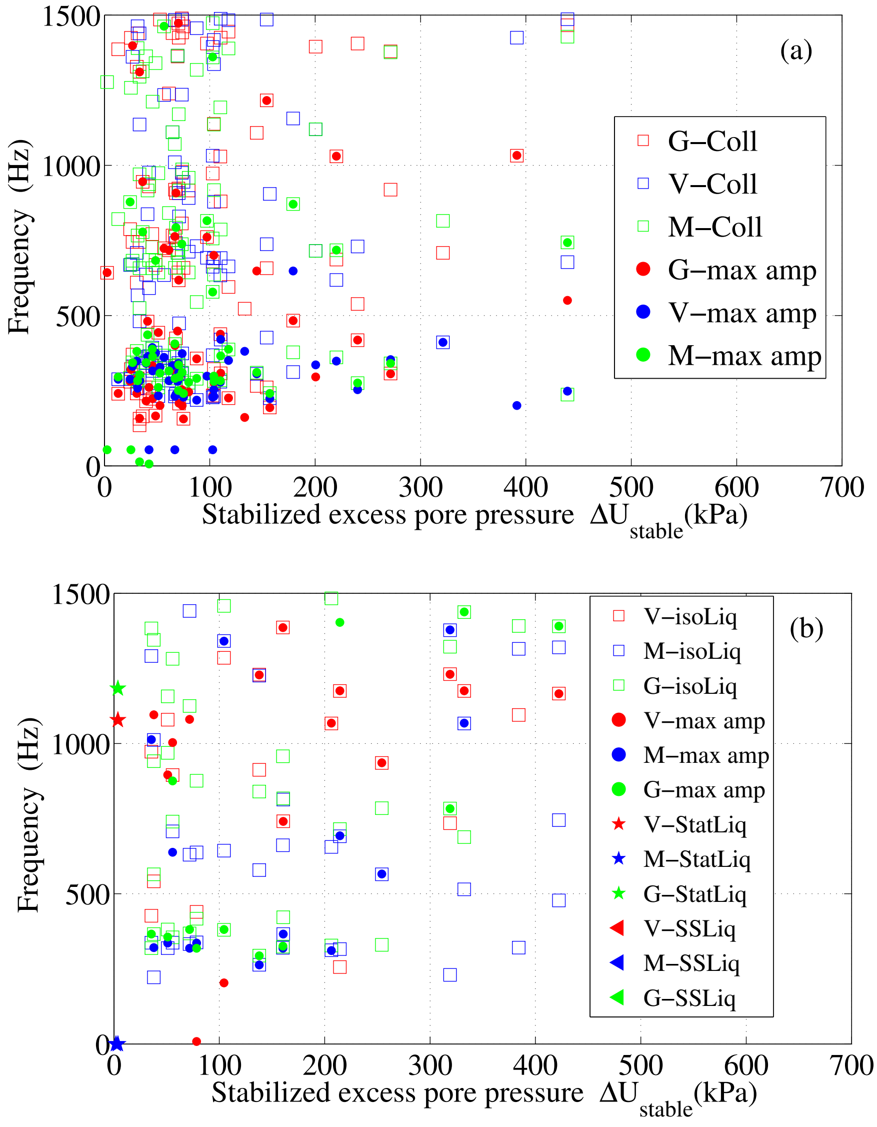

FIG. S5. Characteristic frequencies of collapse (a) and liquefaction (b) identified by CCWT from vertical top cap acceleration G, radial laser vibrometer V and lateral microphone M. SSLiq (StatLiq) means liquefaction from drained (undrained) stick-slip experiment.

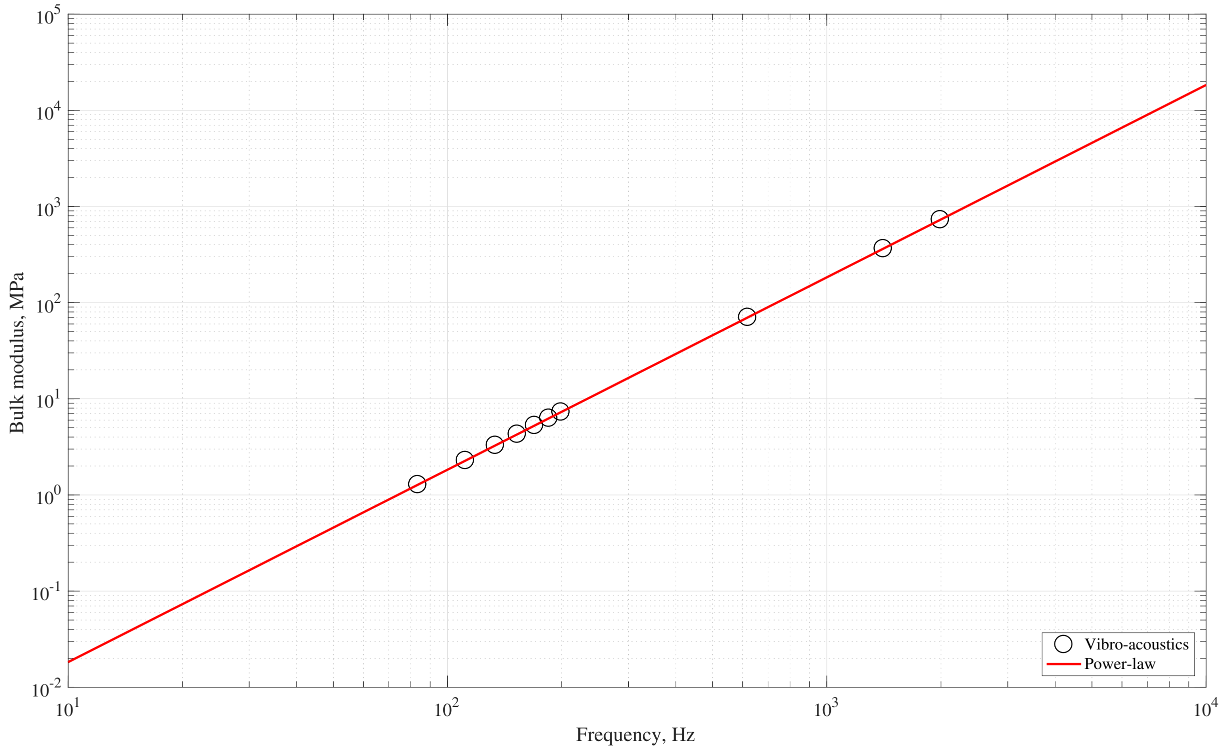

FIG. S6. Power-law relationship between the bulk modulus and the frequency of vibrational modes in numerical simulations with Comsol Multiphysics.

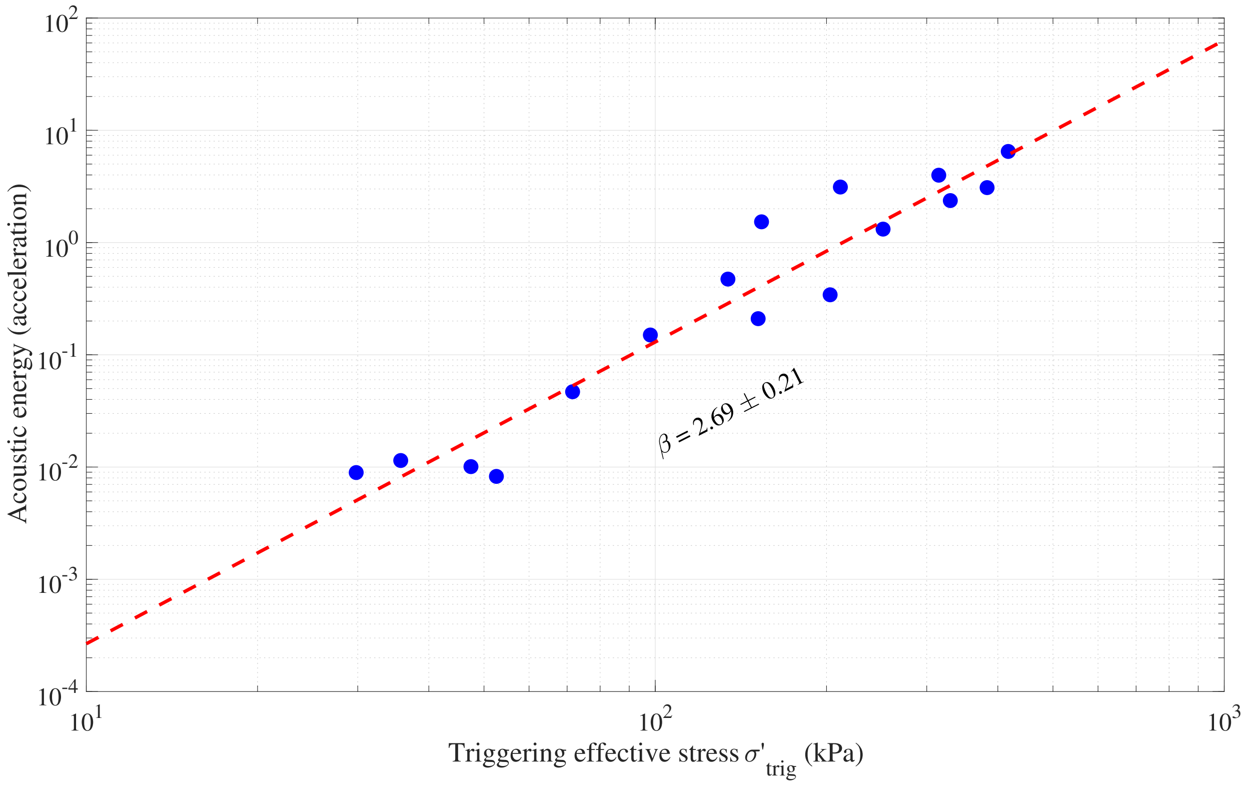

FIG. S7. Power-law relationship with high correlation ($R^2 = 0.922$) between the acoustic energy of the vertical top cap acceleration G and the triggering effective stress from 30 to 418 kPa of uncontrolled triggering stress for isotropic liquefaction failure events.

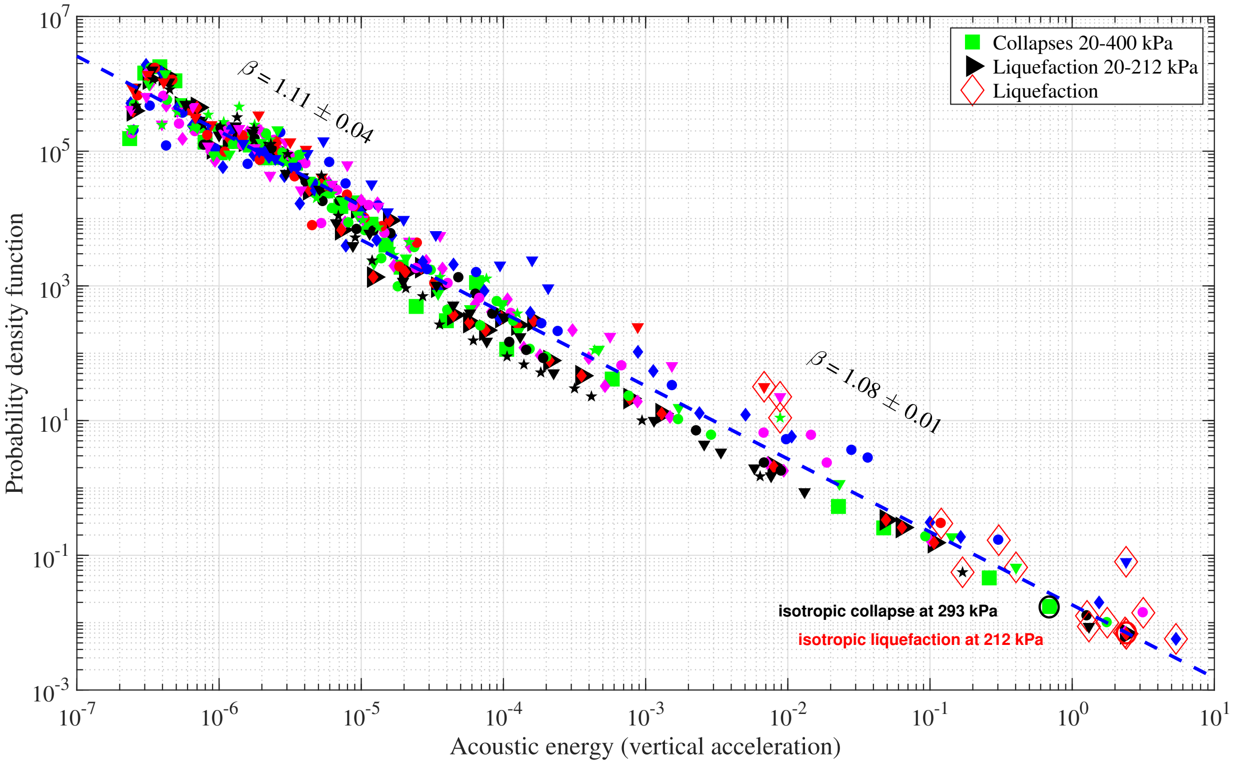

FIG. S8. Double power-law relationship from:acoustic energy with vertical acceleration of the sample top cap : $\beta = 1.11 \pm 0.04$, $R^2 = 0.779$ below $E_c = 1*10^{-5}$ (a.u.) and $\beta = 1.08 \pm 0.01$, $R^2 = 0.951$ beyond E_c. The symbol $\pm$ stands for 95% confidence interval.

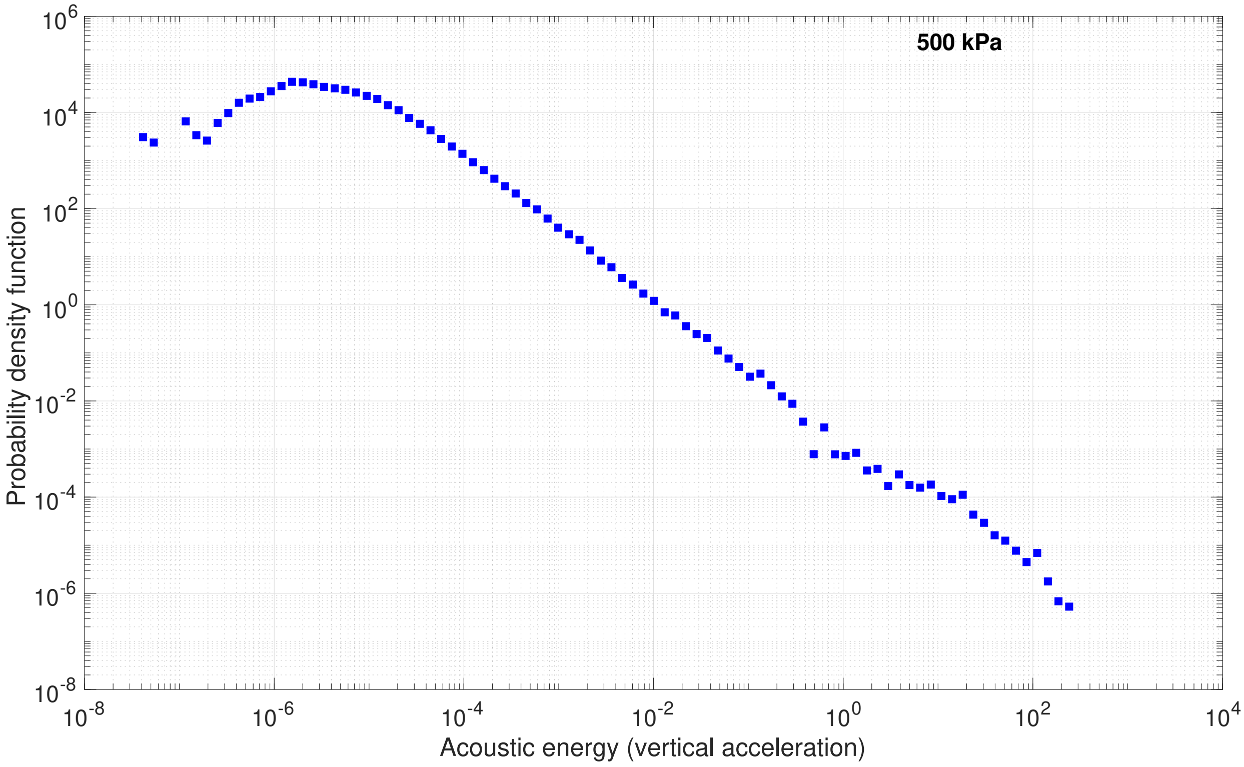

FIG. S9. Power-law relationship with low energy cutoff $E_{min} = 2*10^{-6}$ (a.u.) for water-wet model granular materials in triaxial compression at 500 kPa of normal stress.
